# 3D-SLIP model based dynamic stability strategy for legged robots with impact disturbance rejection

**DOI:** 10.1038/s41598-022-09937-9

**Published:** 2022-04-07

**Authors:** Bin Han, Haoyuan Yi, Zhenyu Xu, Xin Yang, Xin Luo

**Affiliations:** 1 State Key Laboratory of Digital Manufacturing Equipment and Technology, School of Mechanical Science and Engineering, Huazhong University of Science and Technology, Wuhan, 430074 China; 2 Guangdong Intelligent Robotics Institute, Dongguan, 523808 China

**Keywords:** Electrical and electronic engineering, Mechanical engineering

## Abstract

Inspired by biomechanical studies, the spring-loaded inverted pendulum model is an effective behavior model to describe the running movement of animals and legged robots in the sagittal plane. However, when confronted with external lateral disturbances, the model has to move out of the 2-D plane and be extended to 3-D locomotion. With the degree of freedom increasing, the computational complexity is higher and the real-time control is more and more difficult, especially when considering the complex legged model. Here, we construct a control strategy based on the classical Raibert controller for legged locomotion under lateral impact disturbances. This strategy, named 3D-HFC, is composed of three core modules: touchdown angle control, body attitude angle control and energy compensation. The energy loss in each step is taken into consideration, and the real-time measured energy loss of the current step is adopted to predict that of the next step. We demonstrate the efficiency of the proposed control strategy on a simulated 3D-SLIP lower order model and a simulated running quadruped, which are perturbed by different impact forces. Furthermore, a quadruped bionic prototype named MBBOT was set up, on which lateral impact experiments were designed and implemented. Both simulation and experimental results show that the proposed approach can realize the impact disturbance rejection.

## Introduction

It has been well-recognized that legged locomotion has superior performance over its wheeled and tracked counterparts on rough environment due to its discrete footholds, active suspension configuration and larger working space^1^. The past few decades have witnessed a large number of endeavors which were conducted to improve the dynamic performance of legged robots. The spring-loaded inverted pendulum model (SLIP) has been established as a template to approximate the dynamics of trotting, running, and hopping in 2-, 4-, 6-, and 8-legged animals^2,3^. One of the most representative examples employing this idea is the series of monopod and quadruped running machines built by Raibert and his colleagues^4^. After that, many other hopping robots have been built^5–10^, and some legged robots have been designed with the SLIP-like feature^11–15^.

In order to meet the requirements of the real-time control system, different online control methods have been proposed for the SLIP model in the sagittal plane. Raibert provided the first control algorithm for SLIP-based running robots^16^. Koditschek and Buhler introduced two models constrained to hop in one dimension using a simplified Raibert scheme^17^. Taking it one step further, Zeglin and Brown built a planar bow leg monopod robot and controlled it with a combination of graph-searching planning and feedback control algorithm^18^. Saranli et al. proposed control algorithms to make multi-jointed planar monopod hoppers behave like spring-mass systems^19^. Liu presented a control approach for bounding gait of quadruped robots with multi-jointed legs by applying the concept of Virtual Constraints^20^. Nie proposed an open-loop control method including touchdown angle control strategy and leg length control strategy that can drive the quadruped robot with spring legs running in passive bounding gait^21^. Buehler presented the CPDR controller imposing desired trajectories via inverse dynamics to reduce locomotion energy expenditure^22^. The CPDR strategy is able to realize the control of hopping height through energy regulation, but the main concerns of this adjustment are the robot's vertical velocity components. Yu developed a dead-beat controller for the SLIP runner to regulate the apex state based on approximate apex return map^23–25^. He also proposed Sagittal SLIP-anchored task space control for a monopode robot traversing irregular terrain^26^. Schmitt and Clark proposed the AER control strategy based on active energy addition and removal by leg compression and leg extension^27^. Arslan and Saranli realized dead-beat control of hopping machines using three control methods, i.e. the Leg Length Control (LLC), Leg Stiffness Control (LSC) and Two-Phase Stiffness Control (TPSC)^28^. This approach regulated system energy by changing leg length and stiffness. It has certain adaptability to different terrain conditions, but requires relatively long-time numerical computation. Sharbafi proposed a control method for the extended SLIP model based on virtual pendulum posture control to achieve robust hopping against perturbations in the sagittal plane^29^. The authors proposed the Hybrid Feedback Control (HFC) strategy for stability control of SLIP system in sagittal plane on uneven terrain^30^. These methods are suitable for different SLIP-based running robots, but most of them only consider the two-dimensional (2D) planar motion control.

Locomotion in three-dimensional (3D) space is more consistent with the actual situation for a legged running robot, especially in the case that locomotion is bearing lateral impact disturbance. Any lateral impact disturbance upon a legged running locomotion can incur the generation of an unexpected lateral velocity, consequently causing the deviation of the locomotion from its original direction of movement, even falling down to destroy the system. In this case, a 2D planar motion should obviously be extended to the 3D space. In 3D space, the difficulty of algorithms and the computational complexity both increase dramatically compared to the 2D situation.

Most recently, there have been some endeavors to transfer the SLIP model to 3D space. One feasible approach was achieved by feedback control strategy. The best known is that Raibert built 3D monopod hoppers which could be stabilized by feedback control in 1984^31^. Seipel and Holmes derived an approximation to the stride-to-stride Poincaré mapping over a horizontal plane in 3D space, and chose a simple feedback control algorithm to stabilize running motions^32,33^. Another feasible approach was achieved by deadbeat control strategy which could achieve the desired goal in a single step. Carver developed deadbeat control results for a 3D point-mass hopper and analyzed a number of control problems for 3D steering^34^. Wu and Geyer studied 3D-SLIP running and steering time-based deadbeat control laws that provide terrain robustness to the template^35^. Hurst designed the 3D bipedal robot ATRIAS with SLIP-like features and applied a feedback controller to achieve a periodic orbit^36^. Wensing and Orin applied the local deadbeat control to a 3D-SLIP template model, and this method could change speed and recover from disturbances for the system^37,38^. Liu developed a 1-step deadbeat terrain adaptation strategy for humanoid walking based on the 3D Dual-SLIP model^39^. Because of the nonlinear characteristics of the 3D-SLIP model, the analytical solutions cannot be obtained directly. Therefore, deadbeat control laws often require offline/online optimization^40^ or large knowledge bases^34^. However, compared with the lower order models, running robot systems have more complex structures and it is more difficult to get the dynamics approximation in real-time control.

Compared to Raibert's work, the controlled object is expanded from the multi-joint one-legged model to the four-legged model, and the system may be impacted by the unknown disturbances. The complexity of the structure and the external disturbances make it more difficult to control the whole system. Moreover, due to the effects of damping and collision in the actual terrain, accurate energy loss may affect motion stability, especially under lateral impact disturbances. The goal of this paper is to propose a simplified strategy based on 3D-SLIP model to achieve balance control under force impact, and then extend this strategy to the higher order models with more complex structures. This strategy has the advantage of avoiding the complex optimization process for running dynamics. The strategy takes the energy loss in each step into consideration, and the energy loss of the current step is adopted to predict that of the next step. It will provide a simple and effective solution for the real-time control of the multi-legged robot systems.

The remainder of this paper is organized as follows. “Equivalent 3D-SLIP model” section in the methods describes the dynamics of the 3D-SLIP model. “Control method under the impact of disturbance” section  in the methods introduces the control system structure and details of the Three-Dimensional Hybrid Feedback Control (3D-HFC) strategy for the 3D-SLIP stability control and the extension method for the one-legged model and the four-legged model. The simulation results using the 3D-HFC method under the impact of the different situations are shown in “Simulation” section in the results. “Prototype experiment” section in the results shows an impact experiment in the real legged robot prototype named MBBOT. Finally, the conclusions are drawn and the discussions on the future work are made in “Discussion” section.

## Methods

### Equivalent 3D-SLIP model

#### Bionic principle

**Figure 1:**
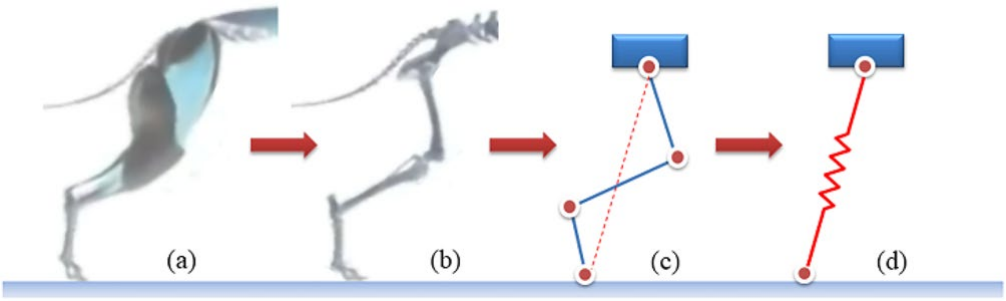
Equivalent process of the single leg. (**a**) Hind leg with muscles. (**b**) Leg bone structure. (**c**) An anchor for the single leg. (**d**) The simplest 2D-SLIP template (the figure is created by Visio 2016, https://support.microsoft.com/visio).

Multiped mammals have very complex leg structure, for example, a dog’s hind leg is composed of femur, tibia, fibula, and hind paw, with muscles, tendons and ligaments providing traction forces for the dog's body. Complicated connection between bones, redundant muscle actuation, and excessive DOFs of joints all lead to high dimension, high computational complexity and high redundancy features of legged locomotion, making its analysis very difficult. Based on observation and analysis of various multipedes running at different speeds, biologists find in legged locomotion the property of elastic energy storage^41,42^, and therefore propose the Spring Loaded Inverted Pendulum (SLIP) model virtually composed of a mass and a leg spring to simplify the description of legged locomotion. The single leg simplification procedure is shown in Fig. 1.

**Figure 2:**
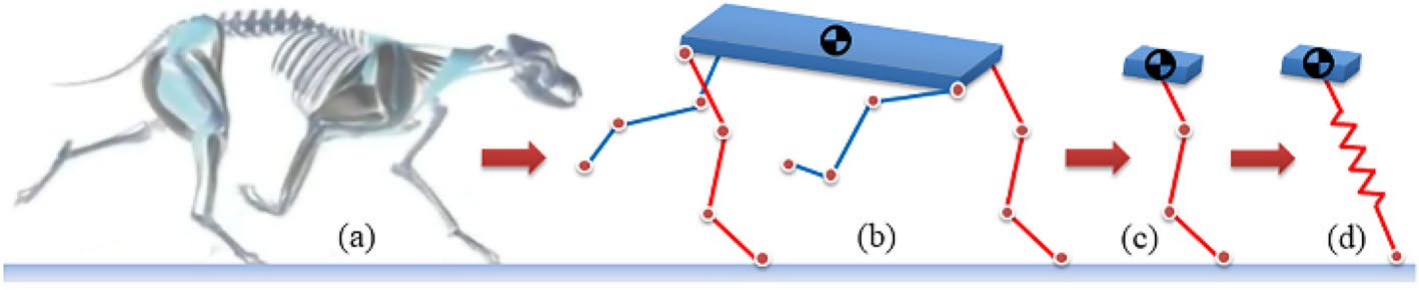
Equivalent process of the quadruped mammal. (**a**) Quadruped mammal. (**b**) Simplified four-legged model. (**c**) An anchor for the equivalent virtual leg. (**d**) The simplest 3D-SLIP template (the figure is created by Visio 2016, https://support.microsoft.com/visio).

Locomotion gaits of multiped mammals can be divided into symmetrical gaits and asymmetrical gaits^43^. Raibert put forward the monopod SLIP model in sagittal plane as the equivalence to rapid symmetric running gait^16^. Take dog’s trotting gait as an example, its diagonal legs have consistent motion phase, touching down on and taking off from the ground at the same time, thus this pair of legs can be equivalently replaced by a single virtual leg at body CoM. The stiffness, force, and torque of the single virtual leg are the synthesis of those of the two diagonal legs. In trotting gait, two pairs of diagonal legs alternate in motion with phase difference to be one half, and the kinetic and dynamic characteristics of these two pairs are symmetric. Therefore, the monopod SLIP model equivalently placed at the body CoM can describe the motion characteristics of quadruped mammals. The establishment of the equivalent virtual leg is shown in Fig. 2.

#### 3D-SLIP model

**Figure 3:**
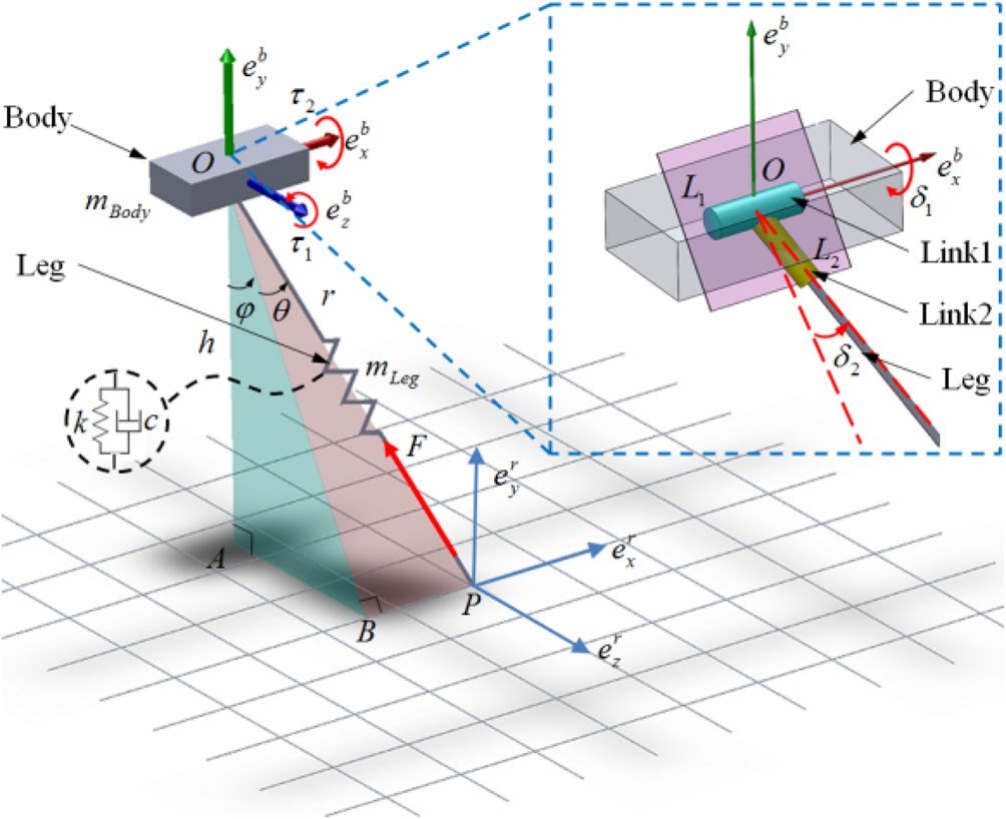
Schematic diagram for the 3D-SLIP model.

Control of the sagittal 2D-SLIP model generally involves only the control of one rotational DOF of the leg spring about the body, and the regulation of body sagittal pitch angle. To extend this model to three-dimensional space, another rotational DOF between body and leg spring is added, and three spatial attitude angles are taken into consideration^44^. The 3D-SLIP model proposed in this paper is shown as Fig. 3.

The 3D-SLIP model consists of a body with lumped mass and equivalent leg spring. in Fig. 3, let point *O* and point *P* denote the equivalent body CoM and the leg toe, respectively. Let $\Sigma {\varvec{e}}^{b}$ be the fixed coordinate system defined at the body’s CoM, and $\left( {{\varvec{e}}_{x}^{b} ,{\varvec{e}}_{y}^{b} ,{\varvec{e}}_{z}^{b} } \right)$ as its base vector. Body and leg spring are connected with a two-DOF rotational hinge at equivalent body CoM. Link 1 has the rotational DOF around base vector ${\varvec{e}}_{x}^{b}$, with rotation angle defined as $\delta_{1}$. Link 2 has the rotational DOF in plane *L*_1_*OL*_2_ with rotation angle defined as $\delta_{2}$. Let $\Sigma {\varvec{e}}^{r}$ be the reference coordinate defined at leg toe, and $\left( {{\varvec{e}}_{x}^{r} ,{\varvec{e}}_{y}^{r} ,{\varvec{e}}_{z}^{r} } \right)$ as its base vector. Body attitude is described with generalized Euler angles $\left( {\alpha ,\beta ,\gamma } \right)$, the sequence of which is a rotation of an angle $\alpha$ about the ${\varvec{e}}_{z}^{b}$ axis, followed by a rotation $\beta$ about the ${\varvec{e}}_{y}^{b}$ axis, and then followed by a rotation $\gamma$ about the ${\varvec{e}}_{x}^{b}$ axis.

*BP*, *OA* and *AB* are the projections of leg spring *OP* on base vectors ${\varvec{e}}_{z}^{r} ,{\varvec{e}}_{y}^{r} ,{\varvec{e}}_{x}^{r}$ respectively. *AOB* is defined as side-swing plane, *BOP* is defined as forward-swing plane. Leg spring attitude is described with side and forward touchdown angles in reference coordinate, namely $\varphi$ in *AOB* and $\theta$ in *BOP*. Equivalent body mass is defined as $m_{body}$, and the equivalent leg spring is considered massless to facilitate subsequent dynamic analysis and calculation. Leg length, equivalent stiffness, and equivalent damping are described with *r*, *k* and *c*. The SLIP model is actuated with three inputs: two torques $\tau_{1}$ and $\tau_{2}$ applied at the hip around z-axis and x-axis respectively and a force *F* acting along the leg.

#### Dynamic analysis of the 3D-SLIP model

**Figure 4:**
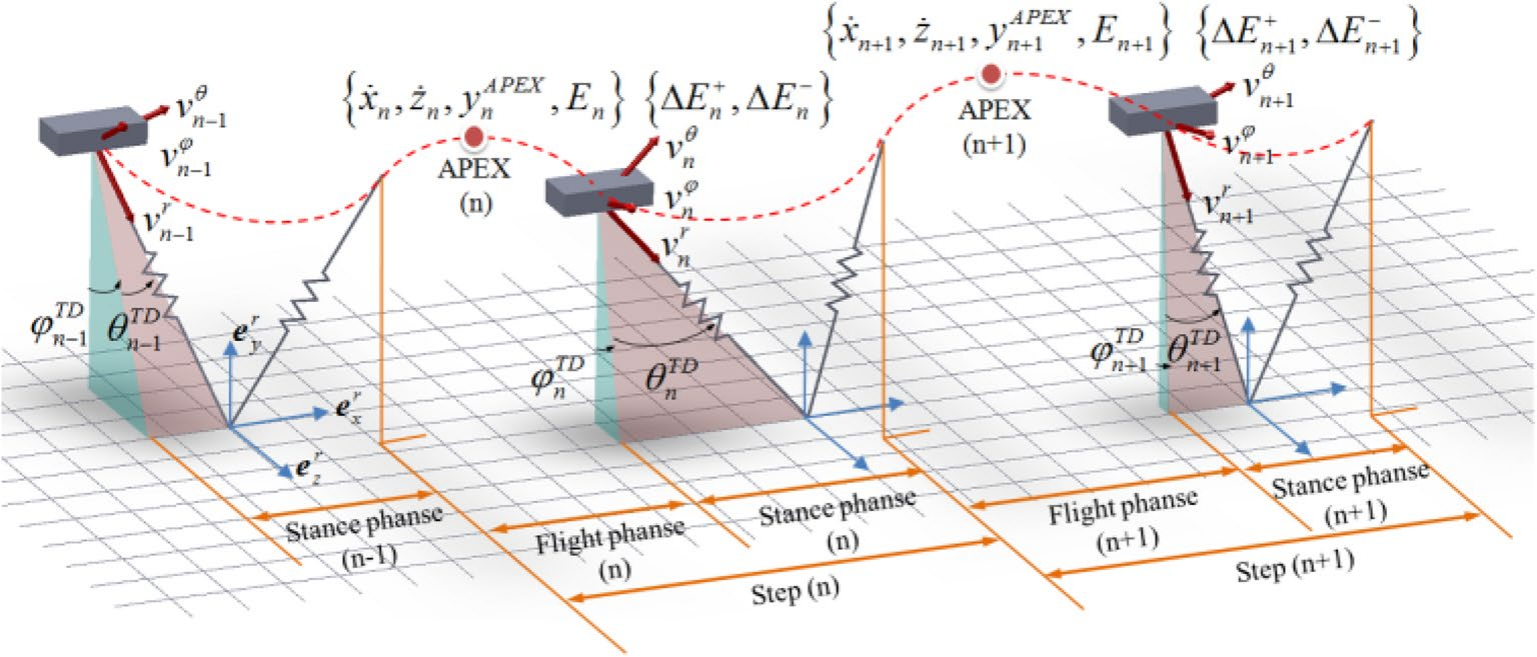
3D-SLIP running period including flight and stance phase in space.

For SLIP model running in sagittal plane, when confronted with a sudden lateral impact force, a velocity perpendicular to this motion plane will be produced, extending the locomotion of SLIP from 2D plane to 3D space. As shown in Fig. 4, in each step, the locomotion of SLIP model in three-dimensional space consists of flight phase and stance phase, with $\varphi^{TD}$ and $\theta^{TD}$ denoting side and forward touchdown angles in TOUCHDOWN event respectively, $\varphi^{LO}$ and $\theta^{LO}$ denoting side and forward liftoff angles in LIFTOFF event respectively. In flight phase, the APEX is triggered when the SLIP reaches its maximum height, where the vertical velocity decreases to zero. States of SLIP can be represented by the characteristic parameters of APEX event in each step, including the horizontal velocity $\dot{x}$, lateral velocity $\dot{z}$, vertical height of body CoM *y*^*APEX*^ and system energy *E*. The apex return map is defined from the current apex variable $U_{n} [\dot{x}_{n} ,\dot{z}_{n} ,y_{n}^{APEX} ,E_{n} ]$ to the next variable $U_{n + 1} [\dot{x}_{n + 1} ,\dot{z}_{n + 1} ,y_{n + 1}^{APEX} ,E_{n + 1} ]$, where *n* represents the number of steps counting from 1.

There are two different dynamic characteristics for the 3D-SLIP model in flight phase and stance phase respectively. Because the system is limited only by the effect of gravity in flight phase, the flight dynamics of the model in Cartesian coordinates can be easily obtained by

1 $$ \left[ {\begin{array}{*{20}l} m & 0 & 0 \\ 0 & m & 0 \\ 0 & 0 & m \\ \end{array} } \right]\left[ {\begin{array}{*{20}c} {\ddot{x}} \\ {\ddot{y}} \\ {\ddot{z}} \\ \end{array} } \right] = \left[ {\begin{array}{*{20}c} 0 \\ { - mg} \\ 0 \\ \end{array} } \right] $$

In stance phase, 3D-SLIP can be seen as an inverted pendulum with leg toe fixed at the ground. We define a polar coordinate system at equivalent body CoM along the direction of leg spring and the direction perpendicular to the leg plane. Body translational velocity can be decomposed into along the direction of leg spring, in leg plane and in side-swing plane perpendicular to the leg. The dynamics equation of stance phase can be derived as

2 $$ \left\{ \begin{aligned} & m\ddot{r} - mr\dot{\theta }^{2} - mr\cos^{2} \theta \cdot \dot{\varphi }^{2} + mg\cos \theta \cos \varphi - k\left( {r_{0} - r} \right) = - c\dot{r} \hfill \\ & \frac{d}{dt}\left( {mr^{2} \dot{\theta }} \right) + mr^{2} \sin \theta \cos \theta \cdot \dot{\varphi }^{2} - mgr\sin \theta \cos \varphi = 0 \hfill \\ & \frac{d}{dt}\left( {mr^{2} \dot{\varphi } \cdot \cos^{2} \theta } \right) - mgr\cos \theta \sin \varphi = 0 \hfill \\ \end{aligned} \right. $$

where *r*_0_ is the original leg length, *r* is the real-time leg length in stance phase.

It is obvious that Eq. (2) is nonlinear differential equations with no exact analytical solutions. In 2D-SLIP cases, various methods have been proposed to search for approximate solutions like the linearization of gravity or using small enough compression ratio of leg spring^23,45,46^. Similar methods can be extended to 3D-SLIP cases, but to obtain solutions with reasonable precision for control with the employment of these methods requires more numerical computation, and will encounter more difficulties in the real-time control. Besides the environmental factors may also affect the real-time control of the system. But the environmental factors can be considered as the external disturbance uniformly. In this paper, a real-time control strategy is developed to realize a 3-D space periodical stable motion control under external impact forces.

### Control method under the impact of disturbance

In previous study, Raibert first proposed a three-part control system to achieve the dynamically stable running for the hopping and quadruped running machines^4^. Based on the Raibert's controller, we have proposed the Hybrid Feedback Control (HFC) strategy for stability control of 2D-SLIP in sagittal plane on uneven terrain^30^. Further, by extending HFC strategy to three-dimensional space and taking into consideration the control of body attitude angles, this paper puts forward a Three-Dimension Hybrid Feedback Control (3D-HFC) strategy for 3D-SLIP model^44^.

#### Control system structure for the 3D-HFC method

**Figure 5:**
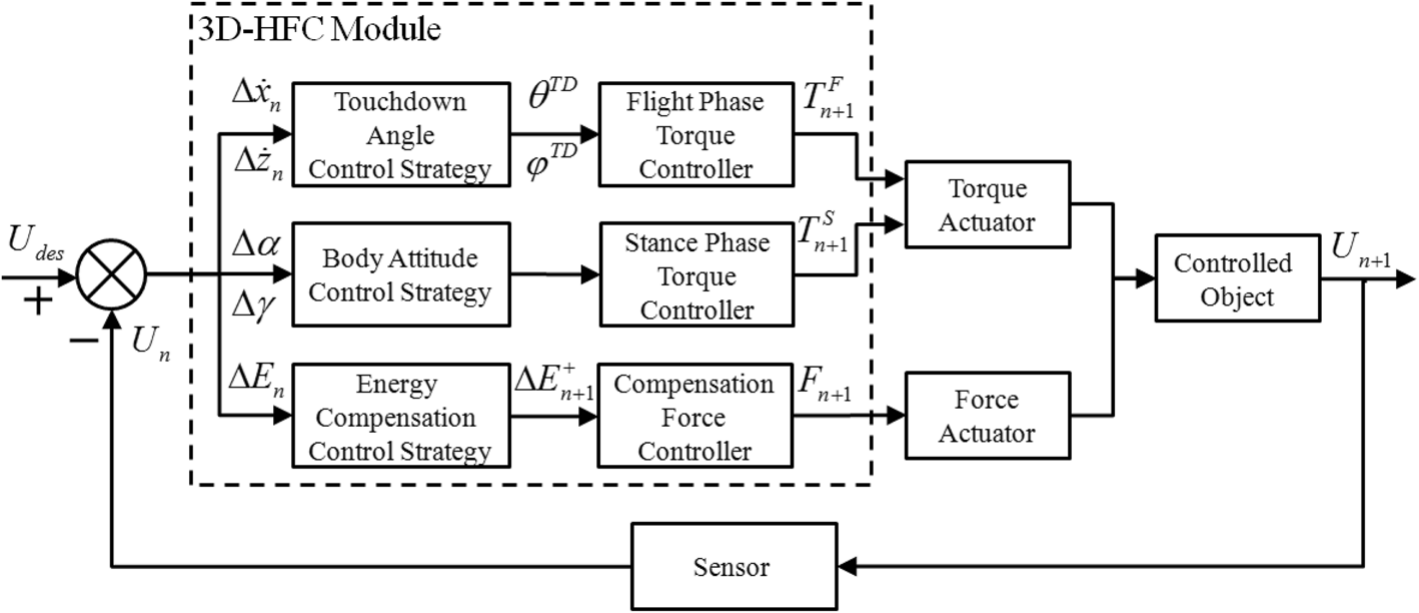
Schematic control system structure of the 3D-HFC method.

Figure 5 shows the control system structure of the 3D-HFC. It is composed of three core modules: touchdown angle control module (TAC), body attitude angle control module (AAC) and energy compensation control module (ECC). Choose $U_{n} [\dot{x}_{n} ,\dot{z}_{n} ,y_{n}^{APEX} ,E_{n} ]$ as the APEX state variable characterizing the state of SLIP system in each step, $U_{des} [\dot{x}_{des} ,\dot{z}_{des} ,y_{des}^{APEX} ,E_{des} ]$ denoting the desired state. The goal of the controller is to get $U_{n}$ as close as possible to the desired state $U_{des}$. The current state $U_{n}$ can be measured through the sensor module at the every apex. Compared with the desired state, the relative error of the state can be obtained and the hybrid feedback control of the SLIP system can be completed through the above-mentioned three control modules.

#### Touchdown angle control strategy

**Figure 6:**
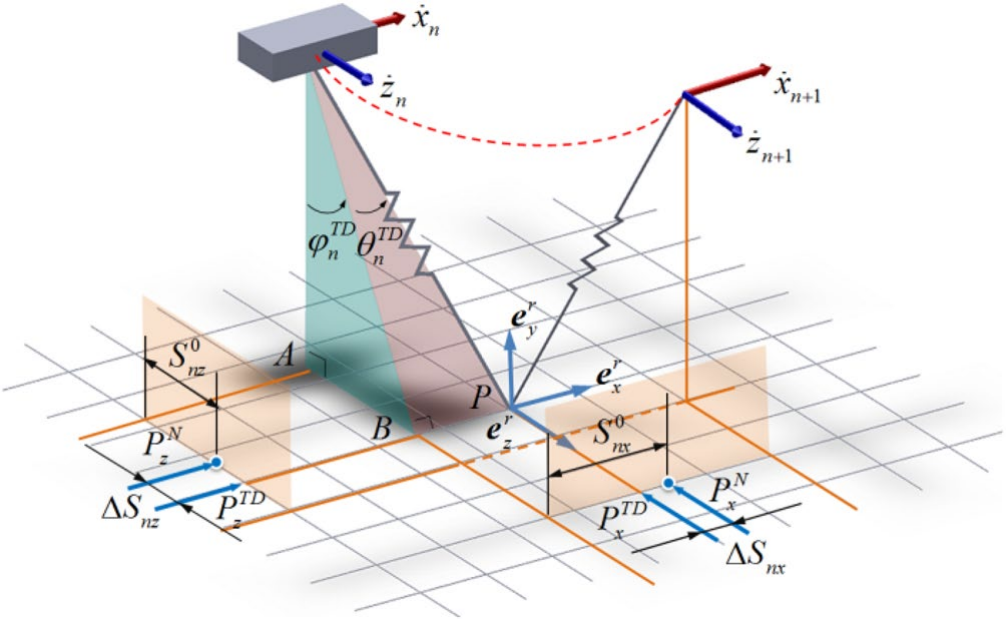
Touchdown angle control strategy for the 3D-SLIP model.

In stance phase, 2D-SLIP can be seen as an inverted pendulum model. With initial conditions determined, this model can realize the periodical jumping from touchdown to liftoff through passive compression and release of the leg spring. 3D space locomotion is similar. Different from the 2D case, we have to control touchdown angles in two directions for 3D-SLIP, $\theta_{n}^{TD}$ in forward-swing plane and $\varphi_{n}^{TD}$ in side-swing plane. 3D-SLIP touchdown angle control is shown in Fig. 6.

The projections of the touchdown point *P* in forward-swing plane and side-swing plane are defined as $P_{x}^{TD}$ and $P_{z}^{TD}$ respectively. The neutral points which have been introduced in Raibert's book^4^ are defined as $P_{x}^{N}$ and $P_{z}^{N}$ in forward-swing plane and side-swing plane. $\Delta S_{nx}$ and $\Delta S_{nz}$ represent distances between projections of touchdown point and neutral point in two planes respectively. According to Raibert’s foot placement algorithms^4^, $\Delta S_{nx}$ and $\Delta S_{nz}$ can be described as follows:

3 $$ \left\{ \begin{aligned} & \Delta S_{nx} = \mu_{1} \left( {\dot{x}_{n} - \dot{x}_{des} } \right) \hfill \\ & \Delta S_{nz} = \mu_{2} \left( {\dot{z}_{n} - \dot{z}_{des} } \right) \hfill \\ \end{aligned} \right. $$

where $\mu_{1}$ and $\mu_{2}$ are feedback gains selected to maximize stability. $S_{nx}^{0}$ and $S_{nz}^{0}$ respectively correspond to the relative position of the neutral points $P_{x}^{N}$ and $P_{z}^{N}$ in two projection planes, and they are half the distances of the horizontal movement of the CoM during the stance phase on the condition it has achieved symmetry movement in two planes. $S_{nx}^{0}$ and $S_{nz}^{0}$ can be obtained through the product of the average speed and the stance phase time, and then they can be determined respectively as

4 $$ \left\{ \begin{aligned} & S_{nx}^{0} = \frac{{\dot{x}_{n - 1}^{avg} \cdot T_{S} }}{2} = \frac{{r_{n - 1}^{TD} \sin \theta_{n - 1}^{TD} - r_{n - 1}^{LO} \sin \theta_{n - 1}^{LO} }}{{2(t_{n - 1}^{LO} - t_{n - 1}^{TD} )}} \cdot \pi \sqrt{\frac{m}{k}}  \hfill  \\ & S_{nz}^{0} = \frac{{\dot{z}_{n - 1}^{avg} \cdot T_{S} }}{2} = \frac{{r_{n - 1}^{TD} \cos \theta_{n - 1}^{TD} \sin \varphi_{n - 1}^{TD} - r_{n - 1}^{LO} \cos \theta_{n - 1}^{LO} \sin \varphi_{n - 1}^{LO} }}{{2 \cdot (t_{n - 1}^{LO} - t_{n - 1}^{TD} )}} \cdot \pi \sqrt{\frac{m}{k}}  \hfill \\ \end{aligned} \right. $$

where $\dot{x}_{n - 1}^{avg}$ and $\dot{z}_{n - 1}^{avg}$ are the average forward speed and the average lateral speed during the stance phase, and $T_{S}$ is the approximate stance phase time^8^.$\left( {r_{n - 1}^{TD} ,\theta_{n - 1}^{TD} ,\varphi_{n - 1}^{TD} ,t_{n - 1}^{TD} } \right)$ and $\left( {r_{n - 1}^{LO} ,\theta_{n - 1}^{LO} ,\varphi_{n - 1}^{LO} ,t_{n - 1}^{LO} } \right)$ represent actual leg length, forward-swing angle, side-swing angle and time at TOUCHDOWN and LIFTOFF phase of the previous stance, respectively. They all can be obtained through the sensing system. Then the forward touchdown angle $\theta_{n}^{TD}$ and side touchdown angle $\varphi_{n}^{TD}$ in Step *n* can be calculated as follows:

5 $$ \left\{ \begin{aligned} & \theta_{n}^{TD} = \arcsin \left( {\left( {S_{nx}^{0} + \Delta S_{nx} } \right)/r_{0} } \right) \hfill \\ & \varphi_{n}^{TD} = \arcsin \left( {\left( {S_{nz}^{0} + \Delta S_{nz} } \right)/\left( {r_{0} \cos \theta_{n}^{TD} } \right)} \right) \hfill \\ \end{aligned} \right. $$

#### Control strategy for body attitude angle

In the simplified SLIP model, the mass of the leg is ignored. With no external forces acting on the system, the body posture remains unchanged during its motion procedure. But in a real platform, the leg mass cannot be set to zero. This leads to a cumulative deviation of the body attitude. Besides, encountered with instantaneous lateral impact forces, body changes its posture. Therefore, the body attitude needs to be regulated in the overall stability control strategy.

Since system angular momentum is conservative in flight phase, the body attitude control could only be realized through the regulation of two driving torques at the hip in stance phase. For model simplification, we assume no slip between the leg toe and the ground in stance phase. The torques controlled to act on the hip around z-axis and x-axis respectively in this phase are determined with linear equations as follows:

6 $$ \left\{ \begin{aligned} & \tau_{1} = - k_{\gamma ,p} (\gamma_{n} - \gamma_{des} ) - k_{\gamma ,v} \dot{\gamma }_{n} \hfill \\ & \tau_{2} = - k_{\alpha ,p} (\alpha_{n} - \alpha_{des} ) - k_{\alpha ,v} \dot{\alpha }_{n} \hfill \\ \end{aligned} \right. $$

where $k_{\alpha ,p}$ and $k_{\gamma ,p}$ are position feedback gains, $k_{\alpha ,v}$ and $k_{\gamma ,v}$ are velocity feedback gains. We can determine the approximate range of these gains based on the simulation results, in order to ensure our first attempt will not damage the system. And then, we can optimize these gains through empirical tuning.

#### Energy compensation control strategy

Previous studies ignore the damping of leg spring in SLIP model and the energy loss in contact between leg toe and the ground. Some researchers take a step further to consider energy loss of SLIP running on flat terrain. However, adding a constant energy to the system for compensation is inapplicable for energy control of system running on rough terrain or under external impact forces. In order to reflect the moving process of practical running systems more precisely, the energy loss in each step is taken into consideration and the real-time measured energy loss of the current step is adopted to predict that of the next step, avoiding the complexity of precise computation. In the Step (n), $\Delta E_{n}^{ + }$. and $\Delta E_{n}^{ - }$ denote the amount of energy added and lost during the stance phase respectively. After the liftoff event, the amount of energy loss $\Delta E_{n}^{ - }$ can be calculated as follows:

7 $$ \Delta E_{n}^{ - } = E_{n}^{TD} + \Delta E_{n}^{ + } - E_{n}^{LO} $$

where $E_{n}^{TD}$ and $E_{n}^{LO}$ are the overall system mechanical energy at the moment of touchdown and liftoff in the Step (n) respectively. They are determined as follows:

8 $$ E_{n}^{TD} = m\left( {(\dot{x}_{n}^{TD} )^{2} + (\dot{y}_{n}^{TD} )^{2} + (\dot{z}_{n}^{TD} )^{2} } \right)/2 + mgr_{n}^{TD} \cos \theta_{n}^{TD} \cos \phi_{n}^{TD} + k(r_{n}^{TD} - r_{0} )^{2} /2 $$

9 $$ E_{n}^{LO} = m\left( {(\dot{x}_{n}^{LO} )^{2} + (\dot{y}_{n}^{LO} )^{2} + (\dot{z}_{n}^{LO} )^{2} } \right)/2 + mgr_{n}^{LO} \cos \theta_{n}^{LO} \cos \phi_{n}^{LO} + k(r_{n}^{LO} - r_{0} )^{2} /2 $$

where $\left( {\dot{x}_{n}^{TD} ,\dot{y}_{n}^{TD} ,\dot{z}_{n}^{TD} ,r_{n}^{TD} ,\theta_{n}^{TD} ,\varphi_{n}^{TD} } \right)$ and $\left( {\dot{x}_{n}^{LO} ,\dot{y}_{n}^{LO} ,\dot{z}_{n}^{LO} ,r_{n}^{LO} ,\theta_{n}^{LO} ,\varphi_{n}^{LO} } \right)$ represent the horizontal velocities, vertical velocities, lateral velocities, leg spring lengths, forward-swing touchdown/liftoff angles and side-swing touchdown/liftoff angles at the touchdown and liftoff moment respectively. These system state variables are obtained through the sensor module in simulation and the Inertial Measurement Units (IMU) on the robot platform.

When the system state converges to the steady-state, the fluctuation of the energy loss gets smaller. Therefore, it assumes that the energy loss of the steady system to be the same as the previous cycle. The amount of compensational energy in the Step (n + 1) can be calculated as

10 $$ \Delta E_{n + 1}^{ + } = \Delta E_{n}^{ - } + E_{des} - E_{n}^{LO} $$

Since the system is energy conservative in flight phase, the system energy should be compensated in the stance phase. Some scholars have proposed different energy compensation strategies like Rated Impulse control (RIC), Constant Force Control (CFC), Variable Stiffness Control (VSC)^23,24^. The VSC method is chosen in this paper because its mechanism is closer to that of practical system. The compensation force of VSC can be determined as

11 $$ F_{n + 1}^{VSC} = \frac{{2 \cdot \Delta E_{n + 1}^{ + } \cdot \left( {r_{0} - r} \right)}}{{\left( {r_{0} - r_{B} } \right)^{2} }} $$

where $r_{B}$ is the minimum spring length, $F_{n + 1}^{VSC}$ acts on the body CoM from bottom to liftoff along the direction of leg spring, the value of which changes according to the real-time leg length.

#### Extension method

**Figure 7:**
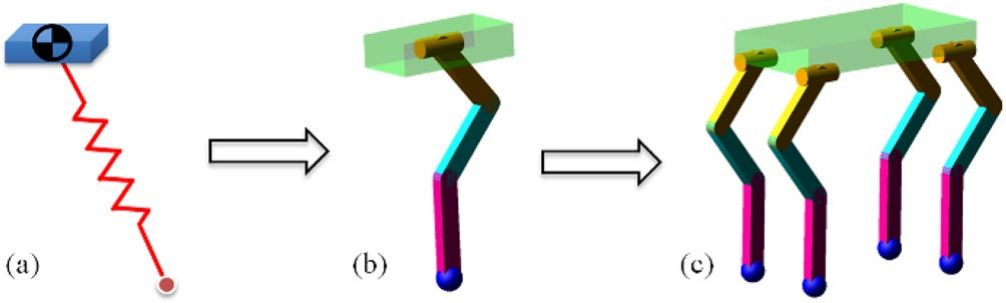
Extension process of the model. (**a**) 3D-SLIP equivalent model. (**b**) Multi-joint one-legged model. (**c**) Four-legged model.

The 3D-HFC method mentioned above is developed assuming a 3D-SLIP equivalent model. However, ultimately, the control is intended for a quadruped robot. Then we are able to extend 3D-HFC approach as follows. First the control strategy for each joint could be chosen and coordinated in a way that achieves the equivalent virtual spring characteristics. Secondly, the multi-joint one-legged model can be expanded to the four-legged model. The control method of each leg is the same as the one-legged model. Then, a variety of gaits can be achieved by alternating motion of each leg. Figure 7 is the extension process of the model.

As the Fig. 7b shown, the multi-joint one-legged model is composed of a mass, three connecting rods and a toe. The one-legged system contains four active control joints, namely the side-swing joint, hip joint, knee joint and ankle joint respectively. In the 3D-SLIP model, only side-swing joint and hip joint require active control. However, knee joint and ankle joint require additional active control in the one-legged model. Four joints should be cooperatively controlled to realize the equivalent virtual spring features.

**Figure 8:**
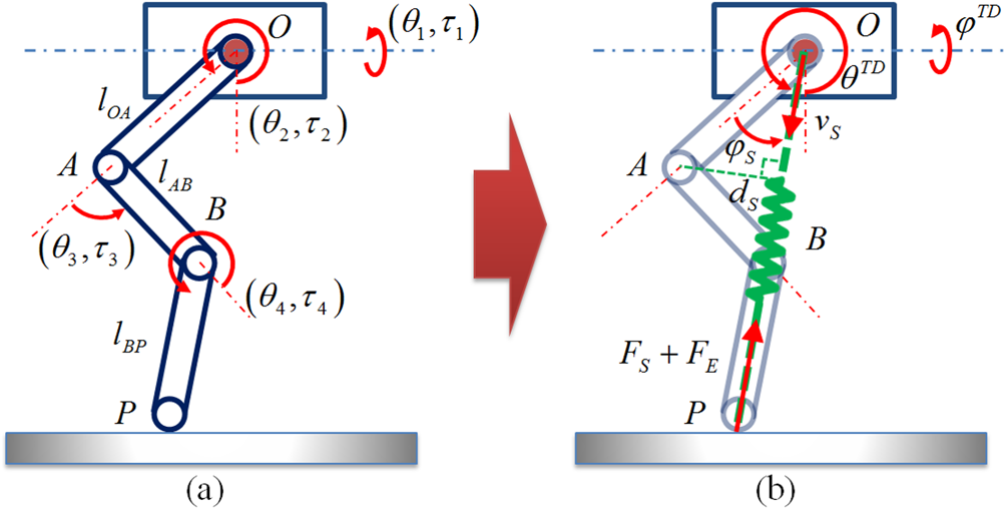
Extended schematic diagram of the one-legged model.

Figure 8 shows the extended schematic diagram of the multi-joint one-legged model. Joint angles and driving torque are defined as $\left( {\theta_{1} ,\theta_{2} ,\theta_{3} ,\theta_{4} } \right)$ and $\left( {\tau_{1} ,\tau_{2} ,\tau_{3} ,\tau_{4} } \right)$ respectively. Points O and P denote the CoM of the body and the foot endpoint. The idea of equivalent extension is to add a virtual spring between the point O and point P. The driving torques of the side-swing joint and hip joint are used to adjust the body posture during the stance phase and control the touchdown angle during the flight phase. The equivalent spring force and compensation force are achieved by torque control of the knee joint. Connecting rod BP is limited to the virtual spring direction through torque control of the ankle joint. The green dashed line between points O and P in the Fig. 8b represents the equivalent virtual leg spring. Its equivalent length, virtual stiffness and viscous damping are defined as $\left( {r_{S} ,k_{S} ,c_{S} } \right)$.

Equivalent extension process is divided into flight phase and stance phase. In the flight phase, the side-swing angle $\varphi^{TD}$ and forward-swing angle $\theta^{TD}$ of the virtual spring correspond to the angles in the side-swing plane and forward-swing plane of the 3D-SLIP model respectively. Theoretical values of these angles can be calculated by the 3D-HFC method in real time from the Eq. (5). The equivalent length of the virtual spring need to keep the original length in the flight phase. The expressions of each joint angle can be obtained through the geometric relationship as follows:

12 $$ \left\{ \begin{aligned} & \theta_{1}^{F} = \varphi^{TD} \hfill \\ & \theta_{2}^{F} = \theta^{TD} - \arccos \left( {\frac{{\left( {r_{S0} - l_{BP} } \right)^{2} + l_{OA}^{2} - l_{AB}^{2} }}{{2l_{OA} \left( {r_{S0} - l_{BP} } \right)}}} \right) \hfill \\ & \theta_{3}^{F} = \arccos \left( {\frac{{\left( {r_{S0} - l_{BP} } \right)^{2} - l_{OA}^{2} - l_{AB}^{2} }}{{2l_{OA} l_{AB} }}} \right) \hfill \\ & \theta_{4}^{F} = - \arccos \left( {\frac{{\left( {r_{S0} - l_{BP} } \right)^{2} - l_{OA}^{2} + l_{AB}^{2} }}{{2l_{AB} \left( {r_{S0} - l_{BP} } \right)}}} \right) \hfill \\ \end{aligned} \right. $$

where $r_{S0}$ represents the original length.

In the stance phase, the connecting rod BP points to the CoM along the direction of the virtual spring. Then the equivalent length $r_{S}$ can be described as follows:

13 $$ r_{S} = l_{BP} + \sqrt {\left( {l_{OA} \sin \theta_{2} + l_{AB} \sin \theta_{3} } \right)^{2} + \left( {l_{OA} \cos \theta_{2} + l_{AB} \cos \theta_{3} } \right)^{2} } $$

Similar to the 3D-SLIP model, equivalent force of the one–legged model also consists of the virtual spring force $F_{S}$ and the compensational force $F_{E}$. $F_{E}$ can be calculated using the Eq. (11) with the 3D-HFC method. Considering the equivalent stiffness and damping, $F_{S}$ can be described as

14 $$ F_{S} = k_{S} \left( {r_{S0} - r_{S} } \right) - c_{S} v_{S} $$

where $v_{S}$ represents the CoM velocity along the direction of the virtual spring. Meanwhile, the body attitude can be adjusted by the side-swing joint and hip joint. The torque expressions are the same as Eq. (6) following the body attitude control strategy of the 3D-HFC method. Then the expressions of each joint torque can be described as follows:

15 $$ \left\{ \begin{aligned} & \tau_{1}^{S} = - k_{\gamma ,p} (\gamma_{n} - \gamma_{des} ) - k_{\gamma ,v} \dot{\gamma }_{n} \hfill \\ & \tau_{2}^{S} = - k_{\alpha ,p} (\alpha_{n} - \alpha_{des} ) - k_{\alpha ,v} \dot{\alpha }_{n} \hfill \\ & \tau_{3}^{S} = \left( {F_{S} + F_{E} } \right) \cdot d_{S} \hfill \\ & \tau_{4}^{S} = - k_{{\theta_{4} ,p}} (\theta_{4} - \theta_{4,des} ) - k_{{\theta_{4} ,v}} (\dot{\theta }_{4} - \dot{\theta }_{4,des} ) \hfill \\ \end{aligned} \right. $$

where $d_{S}$ represents the moment arm between point A and the virtual spring OP, and $\theta_{4,des}$ represents the desired angle which can ensure the rod BP along the direction of the virtual spring. Both of them can be obtained through the geometric relationship as follows:

16 $$ d_{S} = \left| {l_{OA} \cos \varphi_{S} } \right| = \left| {l_{OA} \cdot \frac{{(r_{S0} - l_{BP} )^{2} + l_{OA}^{2} - l_{AB}^{2} }}{{2l_{OA} (r_{S0} - l_{BP} )}}} \right| $$

17 $$ \theta_{4,des}^{{}} = - \arccos \left( {\frac{{(r_{S} - l_{BP} )^{2} + l_{AB}^{2} - l_{OA}^{2} }}{{2l_{AB} (r_{S} - l_{BP} )}}} \right) $$

Equations (12)–(17) provide the extension method from the equivalent model to the multi-joint one-legged model. Further, we can expand the control method to the four-legged model. In the case of the trot gait, four legs of the system can be divided into two groups, and each group includes two legs in a diagonal direction. The motion sequences of two legs in a group are approximately the same. Two groups of legs are usually in a different motion state, one is in the flight phase, and the other one is in the stance phase. Equations (12) and (15) respectively represent the control algorithm in the flight phase and stance phase.

If system body receives the impact disturbance, because of the same motion sequences, one group legs in the flight phase can be seen as an equivalent spring and adjust the touchdown point within the 3D-SLIP algorithm. And another group legs in the stance phase also can be seen as an equivalent spring and provide the compensation force and attitude control torques. Then the extended control methods can be implemented on the four-legged model.

## Results

### Simulation

The ADAMS-Simulink co-simulation technique is employed to verify the validity of the 3D-HFC strategy for 3D-SLIP model under external impact force. There are five different impact situations in Co-simulations: lateral impact when the SLIP system jumps vertically, lateral impact when the SLIP system runs forward, impact along with forwarding motion, compound impact in the running, and lateral impact for extension models. To meet the requirements of the real-time control system, the communication interval between ADAMS and Simulink in all simulations is 0.1 ms^44^.

#### Modeling for simulation

**Figure 9:**
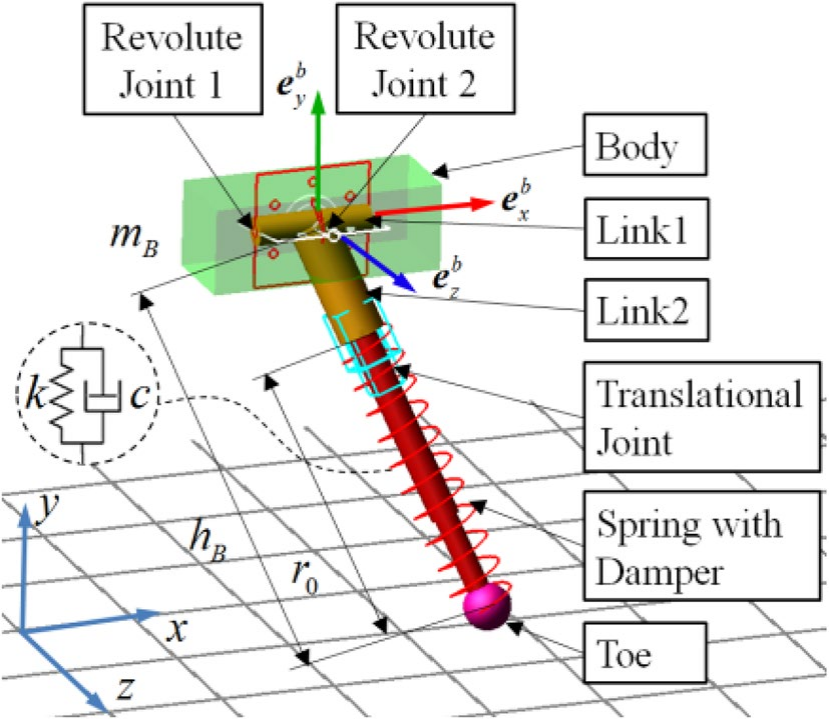
Simulation model of the 3D-SLIP running system in ADAMS.

**Figure 10:**
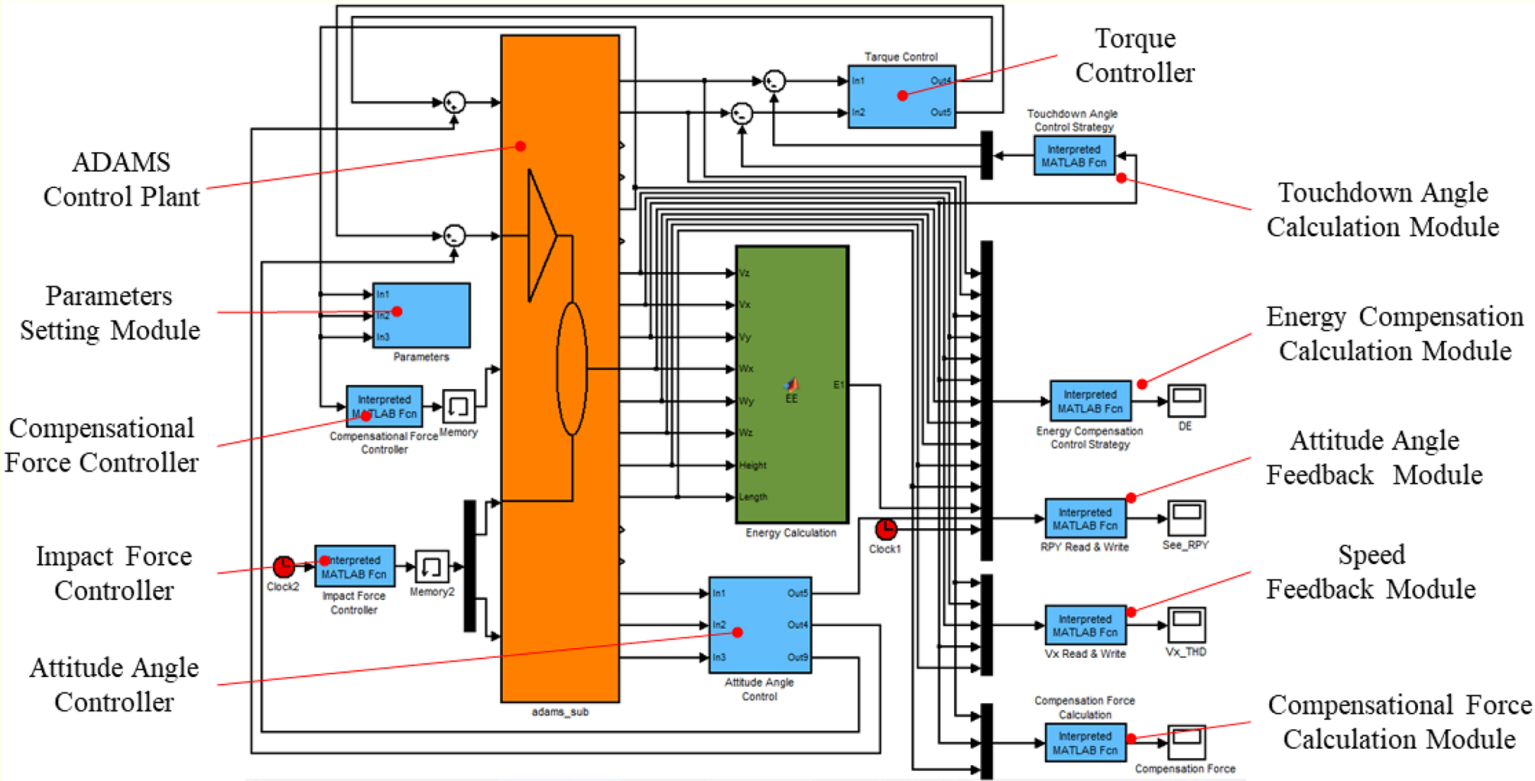
Adams-Simulink co-simulation framework based on 3D-HFC.

The 3D-SLIP running model built in ADAMS is shown in Fig. 9. It includes the body, link 1, link 2, a spring with damper, and a toe. The coordinate systems and angles were defined identically to Fig. 3. The revolute joint 1 was placed between the body and link 1, and the revolute joint 2 was placed between link 1 and link 2. Between link 2 and leg toe, a translational joint was set to limit the movement direction of the leg spring. The compensational force was applied on the body CoM along the spring direction. The contact was defined between the leg toe and the ground. Moreover, a contact sensor was set to judge the touchdown event and liftoff event of the system. Except for the contact sensor, the sensing system also includes the body velocity sensor, spring length sensor, swing angle sensor, etc. The 3D-SLIP model parameters were referenced to the physiological index of physical dogs^47^, which are shown in Table 1. The Adams-Simulink co-simulation framework based on 3D-HFC is shown in Fig. 10.

**Table 1:** Physical parameters for 3D-slip model.

Parameter	Description	Value
*m*_*B*_	Mass of the body	23.6 kg
*m*_*Link1*_	Mass of the link1	0.002 kg
*m*_*Link2*_	Mass of the link2	0.005 kg
*m*_*Toe*_	Mass of the toe	0.003 kg
*h*_*B*_	Distance between toe and COM	0.7 m
*r*_*0*_	Nominal spring length	0.5 m
*k*	Spring stiffness	6 kN/m
*c*	Spring damping	0.01 kNs/m

#### Lateral impact when jumping

**Figure 11:**
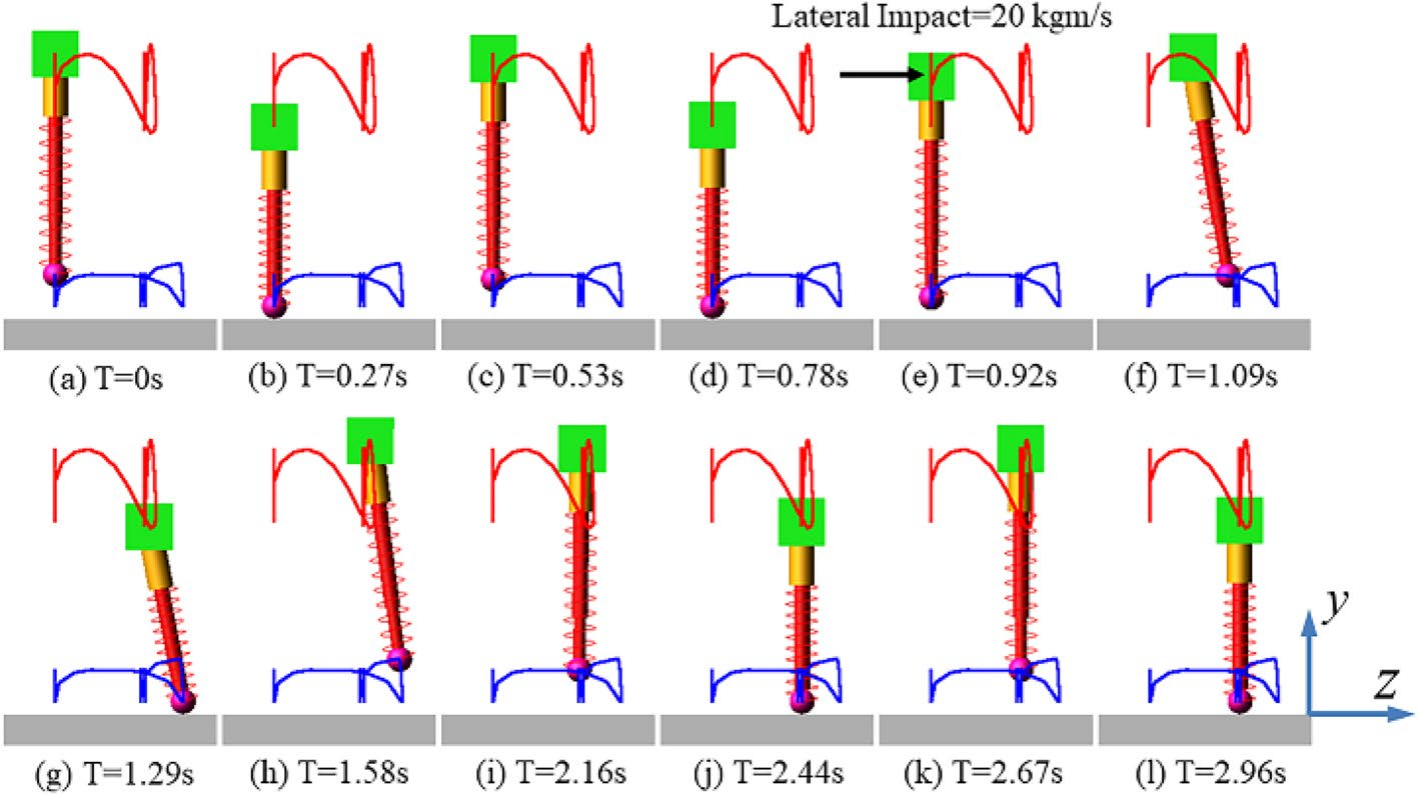
Motion sequence diagram for vertically jumping SLIP system under the lateral impact.

Vertically Simulation 1 (S1) was designed for the lateral impact of the SLIP system during vertical jumps. The initial apex height was set to 840 mm, while the initial forward velocity (along the x-axis) and lateral velocity (along the z-axis) were set to zero. An instantaneous lateral impact was applied on the body CoM along the z-axis at 0.92 s. The impulse value was about 20 kgm/s. The SLIP system did not fall down under the lateral impact. The initial state, including apex height, forward velocity and lateral velocity, was restored using the 3D-HFC method. The motion sequence diagram of this simulation is shown in Fig. 11. The red curve and the blue curve represent the motion trajectories of the body CoM and the toe in the lateral plane, respectively.

**Figure 12:**
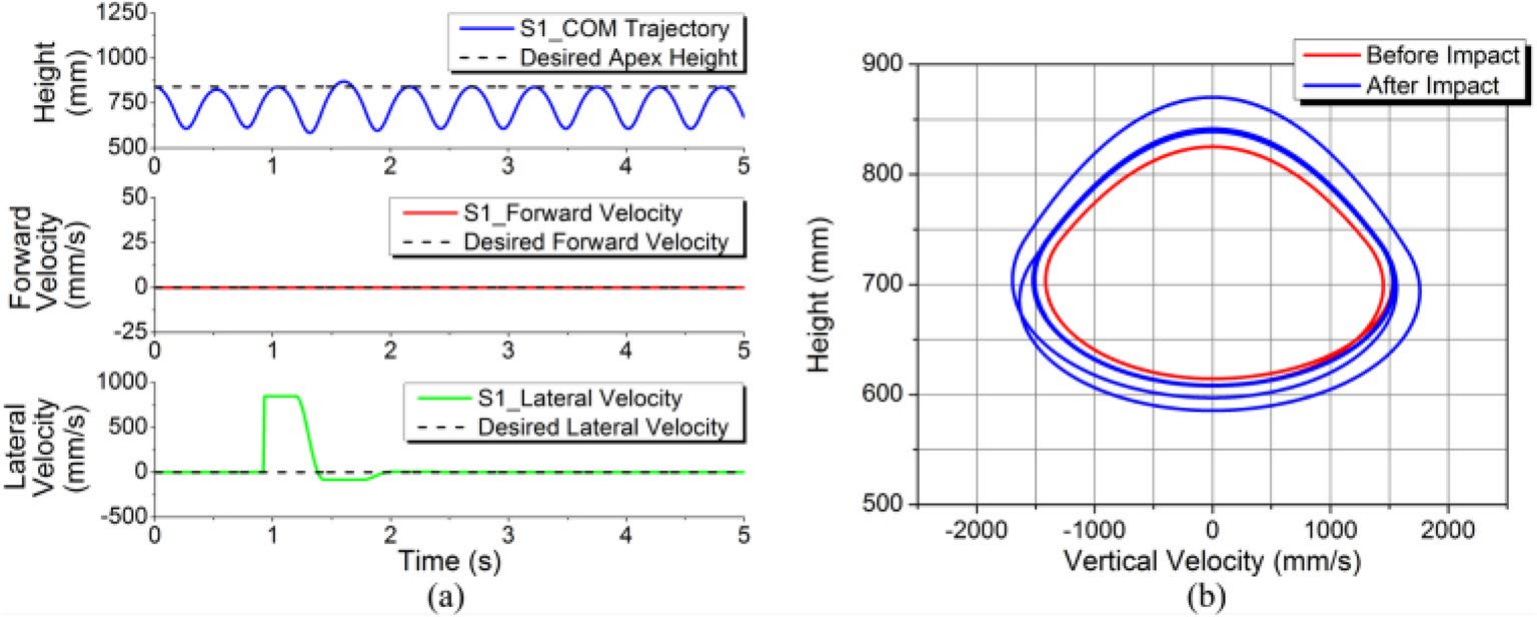
Simulation results for S1. (**a**) Body CoM trajectory, forward and lateral velocities. (**b**) Phase plane trajectory.

Simulation results are shown in Fig. 12. The whole process of the SLIP system achieved the expected goal with the control of the 3D-HFC method according to the height curve (Fig. 12a). Although there is a little fluctuation after impact, the CoM apex height converges to the expected value very quickly. In contrast, the lateral velocity is more obviously affected after the impact. Even though the lateral velocity changed instantaneously, it can still converge rapidly to the desired level in two cycles. Figure 12b is the phase portrait, which shows that the return map can converge to closed curves, indicating the stable periodical motion achieved by the system. It means the system has been controlled at the target state, achieving the desired periodical motion.

#### Impact in running

**Figure 13:**
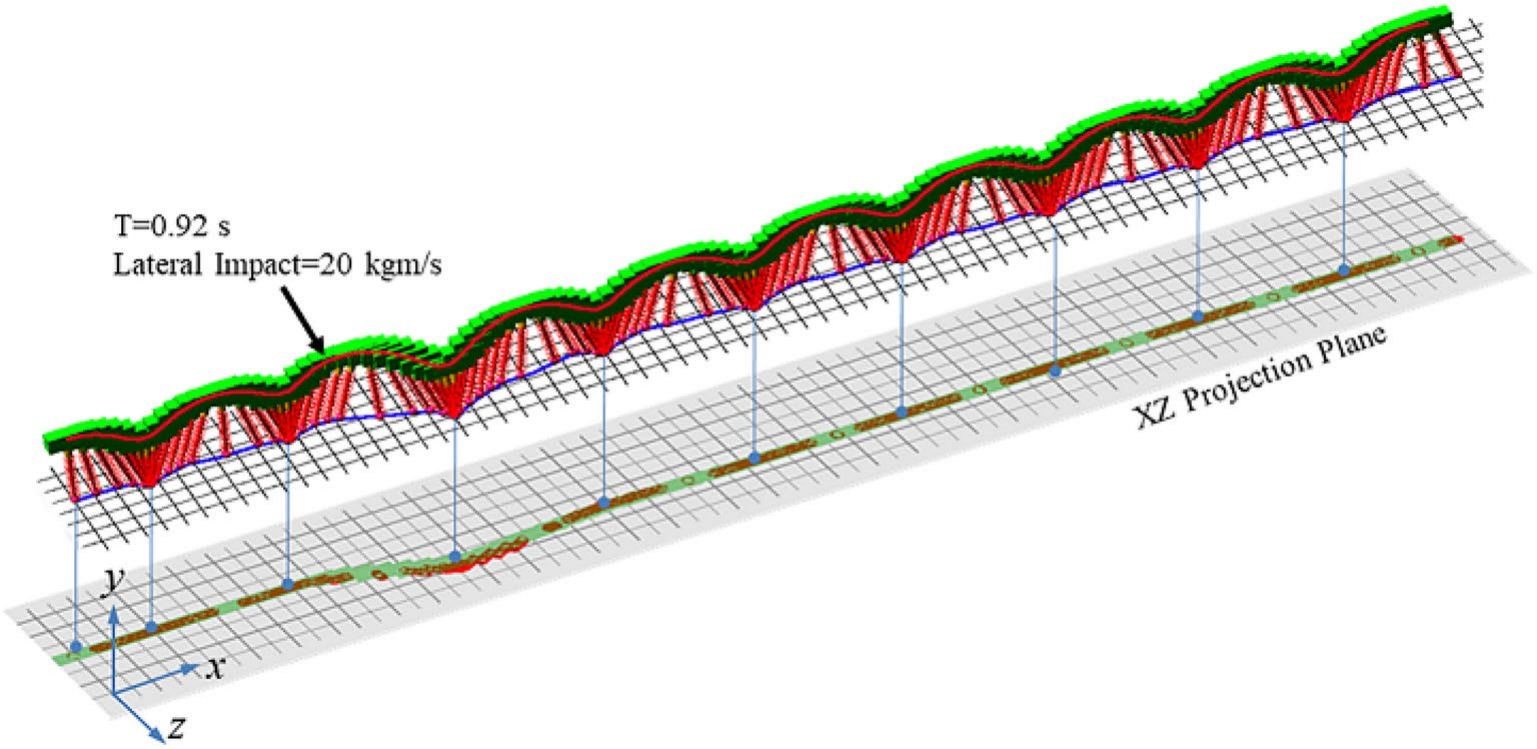
Motion sequence diagram for forward running SLIP system under compound impact.

Simulation 2–4 (S2-S4) was designed for the lateral impact when SLIP system is running forward. In these simulations, the initial forward velocity of the model was set to 2.8 m/s to conduct a running simulation on the flat terrain. The initial apex height and lateral velocity were set to 840 mm and zero, respectively. In S2, an instantaneous lateral impact was applied on the body CoM along the z-axis at 0.92 s, with an impulse value of about 20 kgm/s. In S3, by contrast, the direction of the impact force was changed from the z-axis to the x-axis. Considering the initial forward velocity, the impulse value was reduced to 10 kgm/s. In addition, these above two impacts were simultaneously applied on the body CoM at 0.92 s in S4. The impacts can be seen as different components of a spatial impact, respectively, on the x-axis and z-axis. The value and direction of the spatial impact can be defined by changing the values of the lateral impact and forward impact. The schematic diagram of different impacts is shown in Fig. 13.

The locomotion of SLIP system is extended from 2-D plane to 3-D space as there is a lateral impact. The revolute joint and must be adjusted simultaneously to realize the dynamic stability control in 3-D space. The motion sequence diagram of S4 is shown in Fig. 13. Similarly, the red and blue curves represent the motion trajectories of the body CoM and the toe, respectively. On the X–Z projection plane, it can be clearly seen that the SLIP system deviates from the predetermined direction after impact, but soon recovers stability and continues to move forward along the predetermined direction.

**Figure 14:**
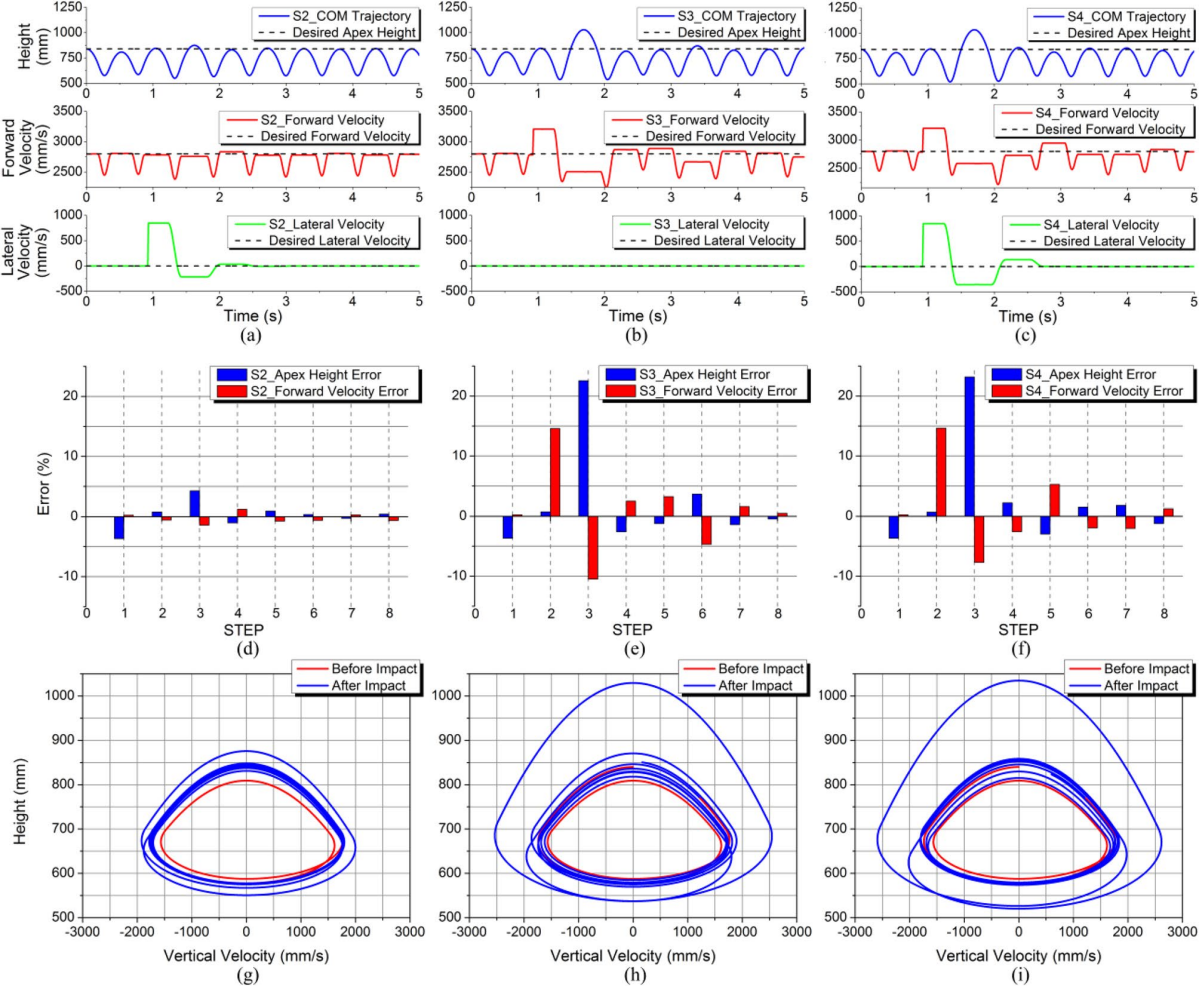
Simulation results for S2–S4. (**a**)–(**c**) Body CoM trajectory, forward and lateral velocities. (**d**)–(**f**) Apex height error and forward velocity error. (**g**)–(**i**) Phase plane trajectory.

The simulation results are shown in Fig. 14. Figure 14a–c show the curves of body CoM trajectory, forward and lateral velocities in S2-S4. It can be seen that the apex height is affected obviously by the impact along forward motion, rather than the lateral impact. And the forward and lateral velocities are affected immediately by forward and lateral impacts respectively. Figure 14d–f show the apex height error and the forward velocity error in each step. When there is only a lateral impact, the errors of apex height and forward velocity are controlled within 5%. And when there is an impact alone the forward motion, apex height error and the forward velocity error can reach about 22% and 15% respectively under the unknown impact. However, the SLIP system will not fall down and can converge quickly to the expected state with an error less than 1% using 3D-HFC method. Figure 14g–i are the phase portraits and it can clearly find that there is a cycle obviously leaving the convergence range after impact. And after few steps, the return map is able to converge to closed curves, indicating the stable periodical motion achieved by the system. S4 is the most complex situation in these simulations, and the SLIP system can still be controlled at the target state and achieve dynamic stability motion. This means that the 3D-HFC control strategy has certain robustness and automatic adaptability, and is able to achieve the control target of the system’s convergence to stability even under a spatial impact.

#### Different impact forces

**Figure 15:**
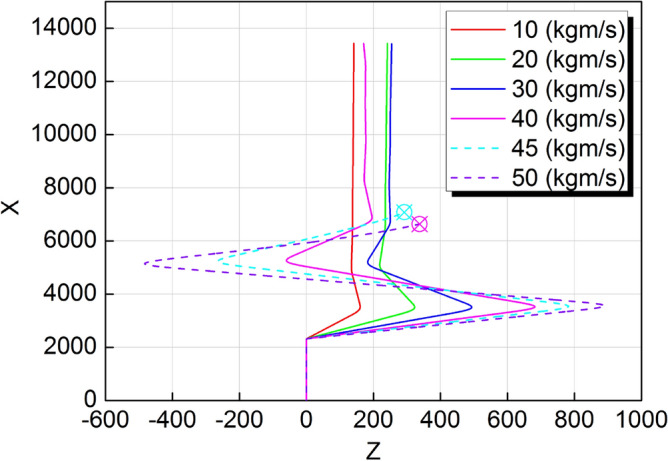
Motion paths in Z-X plane under different impact forces.

**Figure 16:**
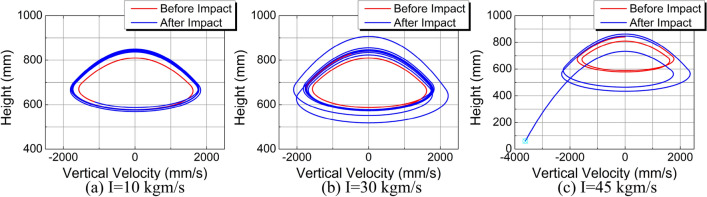
Phase portraits under three different impact forces. (**a**) Phase portraits under the impulse of 10 kgm/s. (**b**) Phase portraits under the impulse of 30 kgm/s. (**c**) Phase portraits under the impulse of 45 kgm/s.

In order to establish the performance limits of the control method, different impact forces were added on the body CoM by scanning. The value of the impulse started with 10 kgm/s, and increased by 10 kgm/s every time. Figure 15 shows the motion paths in Z-X plane under different impact forces. Due to different values of the lateral impact, the lateral velocities after impact are also different. When the impulse is set to 10 kgm/s, the maximum lateral displacement is 160 mm. The system can restore stability within one cycle after the impact and the final offset is 140 mm. When the impulse is set to 30 kgm/s, the system takes two cycles to restore stability, and the final offset increases to 250 mm. However, when the impulse is set to 50 kgm/s, the impact is too large and causes the system to fall after two cycles. This is beyond the capability of the 3D-HFC strategy. After several additional attempts, we found that the stability threshold of the impact was between 44 and 45 kgm/s. Figure 16a–c are the phase portraits under three different impact forces. With the increase of the impulse value, the convergence speed of the system reduces gradually until the system is in a state of divergence. We can more clearly see the effect of the control method from the phase portraits.

#### Lateral impact for extension models

**Figure 17:**
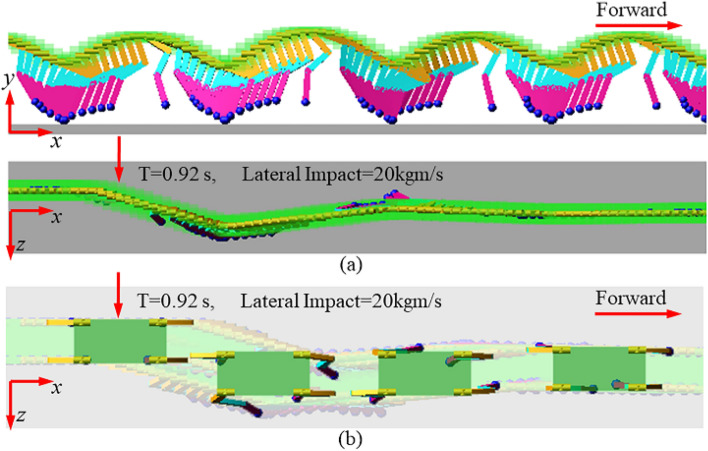
Motion sequence diagrams for extension models under lateral impact. (**a**) Multi-joint one-legged model, (**b**) Four-legged model.

In order to verify the effectiveness of the extension methods, the co-simulations were also performed on the one-legged model and four-legged model. Initial conditions and control targets were the same as the S2. Similarly, a uniform instantaneous lateral impact was added on the body CoM of the 3D-SLIP model, one-legged model and four-legged model respectively. The value of the impulse was about 20 kgm/s. Figure 17a shows the motion sequence diagrams for the one-legged model. In the XY projection plane, the one-legged model can achieve the periodic forward motion similar to the 3D-SLIP model. And in the XZ projection plane, we can also see that the one-legged system deviates from the intended direction after impact, but soon restores stability and continues to move forward along the intended direction. Figure 17b shows the motion sequence diagrams for the four-legged model. The four-legged system can also move forward steadily and periodically within the extension method.

**Figure 18:**
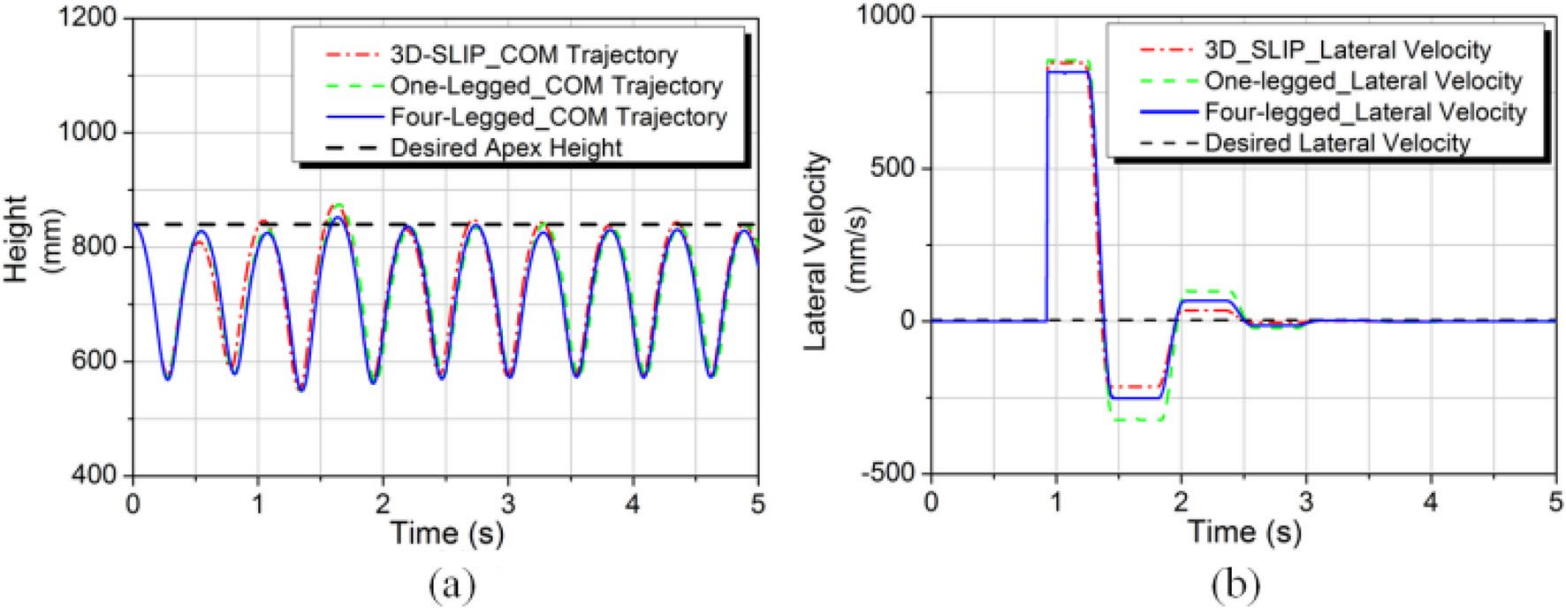
Simulation results of the comparison for different models. (**a**) Body CoM trajectory. (**b**) Lateral velocity.

Figure 18 shows the simulation results of the comparison for different models. Obviously, the characteristics of the CoM trajectory and lateral velocity between three models are quite similar under the lateral impact. This means that the extension method has certain effectiveness for the one-legged model and the four-legged model.

### Prototype experiment

A further goal is to demonstrate the 3D-HFC method on a real legged robot prototype. Although there are certain differences between the equivalent model and the real robot system, the lateral impact experiment is designed for a real robot system to demonstrate and further improve the effectiveness of the balance control method through experimental results.

In order to enhance the performance of quadruped robot, a prototype named MBBOT was developed by a team including researchers from Harbin Institute of Technology (HIT), Huazhong University of Science and Technology (HUST), Shenyang Institute of Automation (SIA), Chinese Academy of Sciences, University of Electronic Science and Technology of China (UESTC) and the Academy of Armored Forces Engineering (AAFE).

**Figure 19:**
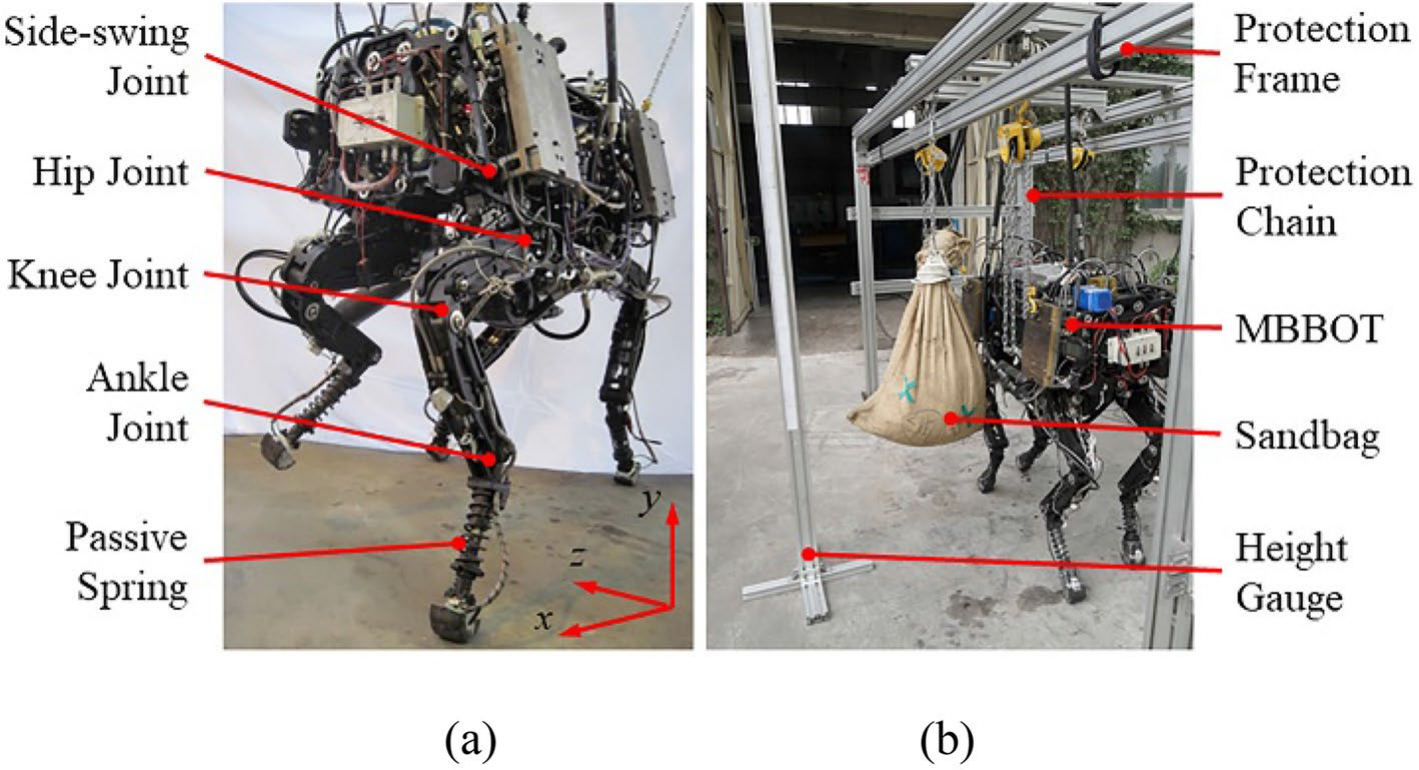
Photos of the experimental prototype and experimental plat-form. (**a**) Experimental prototype named MBBOT. (**b**) Experimental platform for the lateral impact.

In previous literatures, a number of famous quadruped running robots had been introduced, such as the BigDog^48^, SpotMini^49^, HyQ^50^, HyQ2Max^51^, MIT-Cheetah^52,53^, Mini Cheetah^54^, ANYmal^55,56^, KOLT^57^, Laikago^58^, Jueying^59^ and so on. After studying these robot systems, we propose our design for the quadruped platform. As shown in Fig. 19a, the MBBOT consists of a torso module and four leg modules. The torso module is used to fix the power system and central control unit, and to connect the four leg modules as a platform. Each leg module contains four active rotary joints and a passive spring, and has four active DOFs and a passive DOF. The four active rotary joints correspond to the Side-swing Joint, Hip Joint, Knee Joint and Ankle Joint respectively, and they are driven by identical hydraulic cylinders. All 16 joint-actuators are powered by the external hydraulic pump which is also fixed on the torso module. There are 41 sensors on the robot prototype, such as displacement sensors and load cells on the hydraulic cylinders and springs, 3-component force sensors in feet and an Inertial Measurement Units (IMU) on torso. The controller of the MBBOT is the PC-3363 bus based on PC/104. It is a small size, low power consumption, superior performance single board computer (SBC) with built-in floating point operation unit (FPU). PC-3363 board adopts the stacking structure, so it is very convenient to expand function modules. The details of the controller are shown in Table 2. The total weight of the MBBOT is about 150 kg, and the mass ratio between torso and single leg is about 10:1.

**Table 2:** Parameters of the controller.

Processor system	CPU	Intel Atom D525
	Frequency	1.8 GHz
Memory	The maximum capacity	1 GB
	VRAM	Share system memory to 224 MB
I/O	Serial port	Two RS232 ports, one RS485/RS422 multi-functional port
	Keyboard interface	1
	GPIO	8-bit universal I/O
	I2C	1

**Figure 20:**
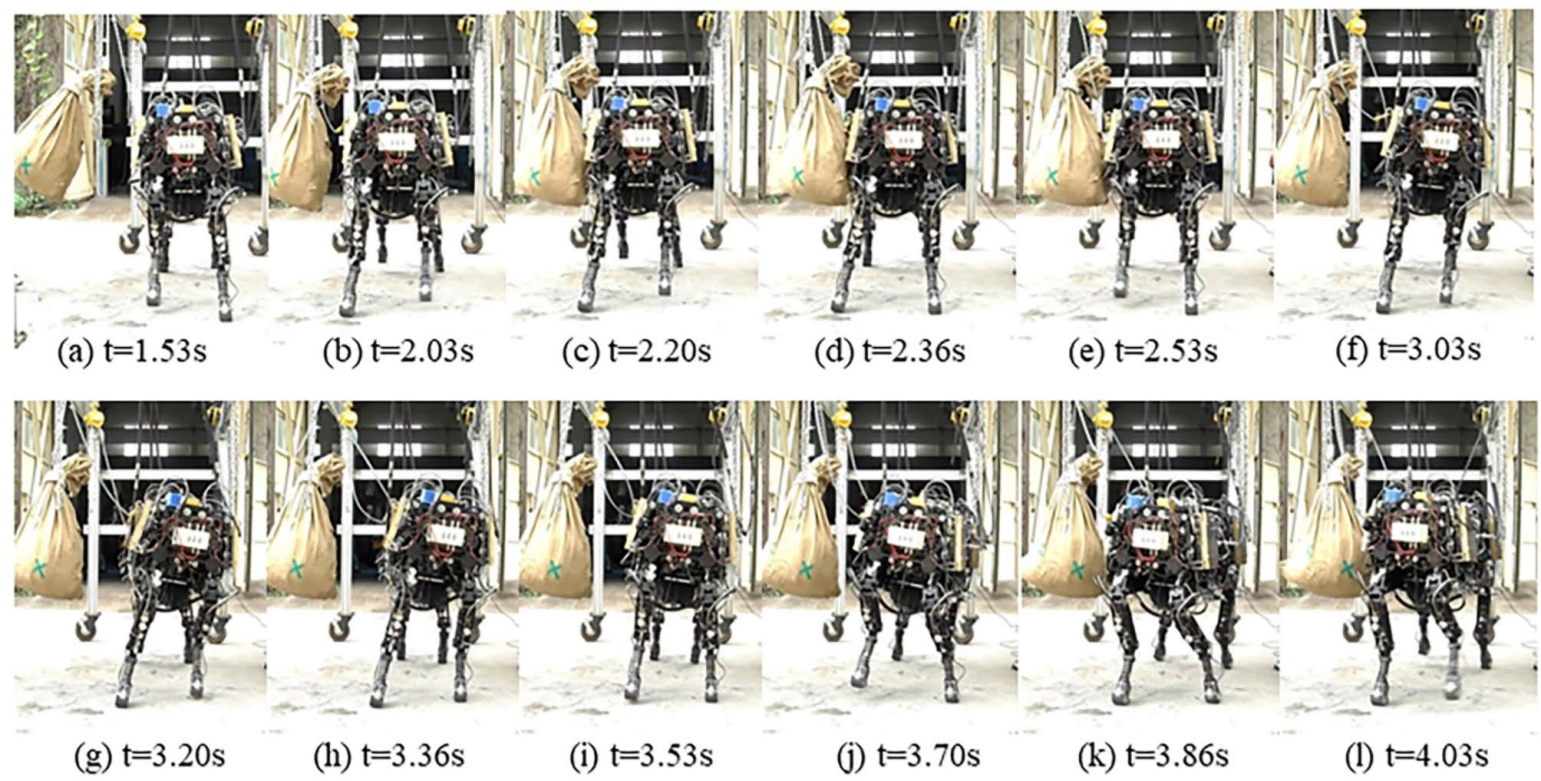
Sequence of photographs showing the complete process of the lateral impact experiment.

The lateral impact experiment platform is shown in Fig. 19b. A sandbag weighing about 40 kg is hung on the bracing frame. The lateral impact force is generated by the sandbag as a swinging pendulum. The sandbag impacts the MBBOT at the lowest swing point and the impact value can be estimated by the relative vertical distance between the initial swing point and the lowest swing point. For example, the relative vertical distance is about 0.11 m in the lateral impact experiment, and the instantaneous impulse value can reach 60 kgm/s. Protection frame is used to prevent the robot falling down if the control algorithm fails during the lateral impact experiment. The action sequence diagram of the impact experiment is shown in Fig. 20.

**Figure 21:**
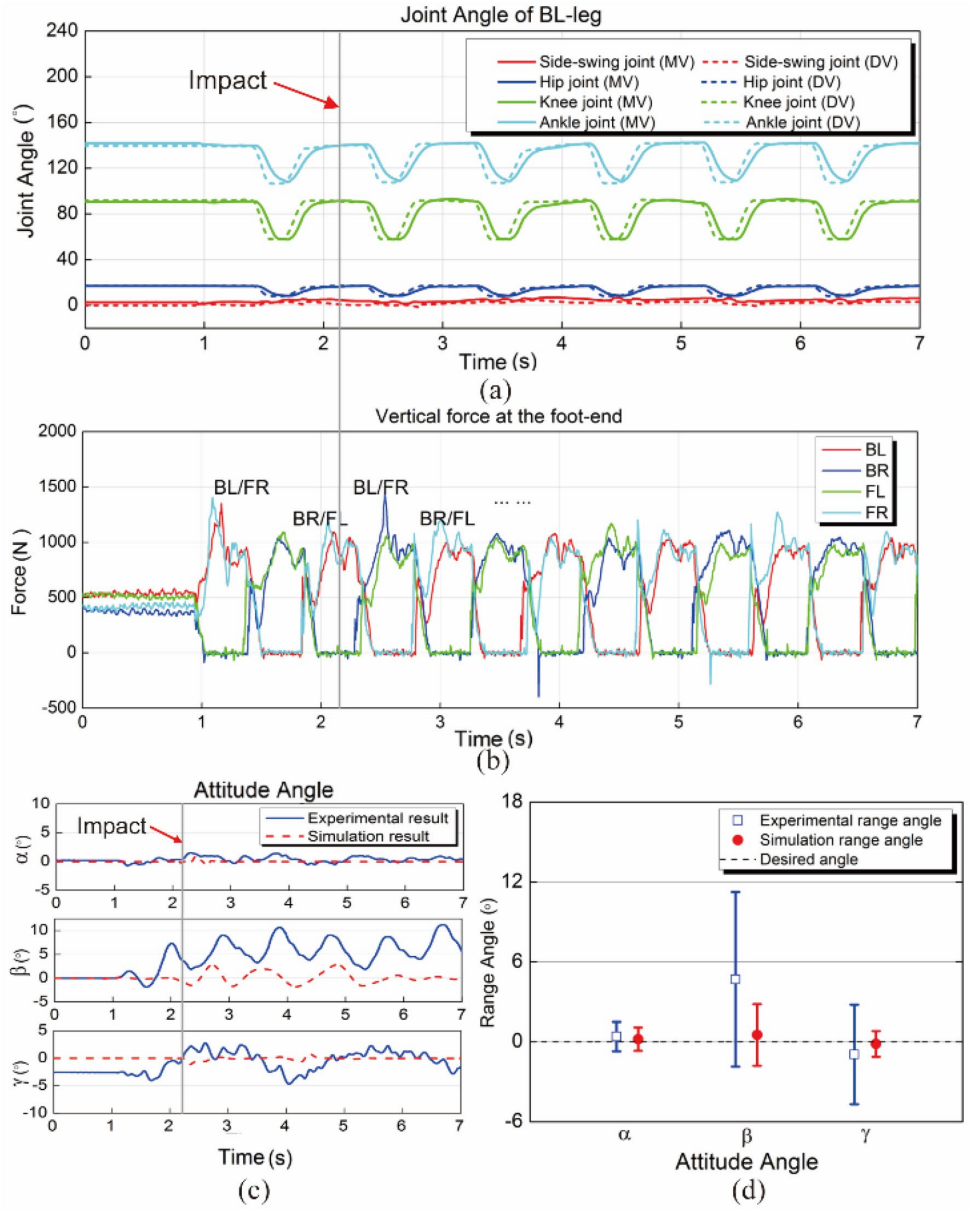
Experimental results. (**a**) Joint angle curves of the front-left leg. (**b**) Vertical force at the foot-end. (**c**) Attitude angles under the lateral impact. (**d**) Range of the attitude angles.

The experimental results are shown in Fig. 21. The initial state of the MBBOT is stepping there waiting for the impact from the swinging sandbag. A moment later, the sandbag swings to the lowest point and impacts on the MBBOT. The IMU is detecting the body attitude angles and the change of the acceleration, and then the planning joint angles can be calculated by the extension method based on the 3D-HFC algorithm. Figure 21a is the joint angles of the back-left leg (BL-leg) during the impact progress. Solid lines and dotted lines represent the measured values (MV) and the desired value (DV). Figure 21b shows the vertical force at all legs foot-end for each duration. During the stance phase, the vertical force is high. And in contrast, vertical force is low during the flight phase. In the graph, the gait-pattern shows obvious symmetric characteristics. The attitude angles are shown in the Fig. 21c. We can see that the attitude angle $\gamma$ is most affected by the lateral impact and the maximum deflection angle can reach $11^\circ$. And Fig. 21d shows the range of the attitude angle for the experiment and simulation. By the analysis of the results, attitude angles in simulation oscillated around the desired angle and had a smaller fluctuation range. The maximum angular deviation was about $2.8^\circ$. In contrast, attitude angles in experiment more deviated from the desired angle, and had a greater fluctuation range. The maximum angular deviation was about ${11}{\text{.1}}^\circ$. The differences of control effect may be caused by the leg quality of the prototype robot. Another possible reason was that the sandbag didn't precisely impact on the CoM of the prototype. However, the MBBOT could restore stability after the impact with the extension method. This experiment verifies the feasibility and effectiveness of the extension based on 3D-HFC algorithm under the instantaneous lateral impact.

## Discussion

A dynamic stability control method is proposed for SLIP running systems in 3D space in this study. In case of an unknown instantaneous force impact, the SLIP system is able to quickly restore stability without falling from impact disturbance. As a result of hybrid feedback control, the 3D-HFC method has a strong ability of fault tolerance control and environmental adaptation performance. The energy loss in each step is taken into consideration in the controller to improve the stability. The co-simulations verify the good control effect of system under lateral impact when jumping vertically, lateral impact when running forward, impact along forward motion and compound impact in running. This method has also been extended to apply on the real legged robot platform, and a lateral impact experiment has been designed and implemented.

Since the ideal equivalent SLIP model ignores the mass of the leg, the spatial rotational kinetic energy is not taken into account in the theoretical dynamic model. However, the leg mass is not zero in the real system, and the body attitude angle will change with the motion of the leg. This is the reason why the 3D-HFC method increases the body attitude control module. Fortunately, the mass ratio between body and single leg is about 10:1, the influence of the mass and inertia of the leg can be reduced or eliminated by the body attitude adjustment control. And the extension method also could be used in the real robot prototype. But in the further work, the improved dynamic model will be intended to establish considering the mass and inertia of the leg.

Constrained by the form of the impact, the lateral impact experiment was not conducted in running. In the future work, an improved way will be attempted to find to generate the lateral impact which could be accurately measured in the process of robot movement. We also hope to extend this method to uneven terrains. More experiments with different impact values also will be designed and implemented on the MBBOT prototype in order to perfect and optimize the 3D-HFC control algorithms.
